# Improvement of Lung Function by Micronutrient Supplementation in Patients with COPD: A Systematic Review and Meta-Analysis

**DOI:** 10.3390/nu16071028

**Published:** 2024-04-01

**Authors:** Mingxin Li, Liangjie Zhao, Chenchen Hu, Yue Li, Yang Yang, Xiaoqi Zhang, Quanguo Li, Aiguo Ma, Jing Cai

**Affiliations:** 1 School of Public Health, Qingdao University, Qingdao 266000, China; limingxin@qdu.edu.cn (M.L.); 2021024467@qdu.edu.cn (L.Z.); huchenchen@qdu.edu.cn (C.H.); 2018010058@qdu.edu.cn (Y.Y.); magfood@qdu.edu.cn (A.M.); 2 Endemic and Parasitic Diseases Prevention and Control Division, Binzhou Centre for Disease Prevention and Control, Binzhou 256600, China; liy_moon@163.com; 3 Institute of Nutrition and Health, Qingdao University, Qingdao 266000, China; 4 Department of Respiratory, Weifang No. 2 People’s Hospital, Weifang 261000, China; xiaoqizhang2024@163.cm (X.Z.); liquanguo2008@163.com (Q.L.)

**Keywords:** micronutrients, chronic obstructive pulmonary disease, randomized controlled trial, meta-analysis, lung function

## Abstract

Background: A healthy, well-balanced diet plays an essential role in respiratory diseases. Since micronutrient deficiency is relatively common in patients with chronic obstructive pulmonary disease (COPD), micronutrient supplementation might have the beneficial health effects in those patients. This systematic review and meta-analysis aimed to demonstrate the impact of micronutrient supplementation on the lung function of patients with COPD. Methods: The PubMed, Cochrane Library, and Web of Science databases were searched from their corresponding creation until February 2024. Search terms included ‘chronic obstructive pulmonary disease’, ‘COPD’, ‘micronutrients’, ‘dietary supplements’, ‘vitamins’, ‘minerals’, and ‘randomized controlled trials’. Meta-analysis was performed to evaluate the effects of micronutrient supplementation alone or complex on lung function in patients with COPD. Results: A total of 43 RCTs fulfilled the inclusion criteria of this study. Meta-analysis revealed that vitamin D supplementation could significantly improve FEV1% (WMD_differences between baseline and post-intervention (de)_: 6.39, 95% CI: 4.59, 8.18, *p* < 0.01; WMD_post-intervention indicators (af)_: 7.55, 95% CI: 5.86, 9.24, *p* < 0.01) and FEV1/FVC% (WMD_de_: 6.88, 95%CI: 2.11, 11.65, WMD_af_: 7.64, 95% CI: 3.18, 12.10, *p* < 0.001), decrease the odds of acute exacerbations, and improve the level of T-cell subsets, including CD3^+^%, CD4^+^%, CD8^+^%, and CD4^+^/CD8^+^% (all *p* < 0.01). The effects of compound nutrients intervention were effective in improving FEV1% (WMD_de_: 8.38, 95%CI: 1.89, 14.87, WMD_af_: 7.07, 95%CI: −0.34, 14.48) and FEV1/FVC% (WMD_de_: 7.58, 95% CI: 4.86, 10.29, WMD_af_: 6.00, 95% CI: 3.19, 8.81). However, vitamin C and vitamin E supplementation alone had no significant effects on lung function (*p* > 0.05). Conclusions: Micronutrient supplementation, such as vitamin D alone and compound nutrients, has improved effect on the lung function of patients with COPD. Therefore, proper supplementation with micronutrients would be beneficial to stabilize the condition and restore ventilation function for COPD patients.

## 1. Introduction

Chronic obstructive pulmonary disease (COPD) has become the world’s third leading cause of death [1]. In 2019, more than 200 million patients with COPD were reported globally [2]. It is a heterogeneous lung disease with persistent respiratory symptoms and airflow obstruction caused by abnormalities in the airways and/or alveoli [3]. The patients will present with a series of symptoms such as cough, sputum, dyspnea, and acute exacerbation due to decreased immune function [4]. Lung function is an important indicator to evaluate the degree of disease in patients with COPD [5]. In other lung diseases, the number of T lymphocytes was significantly correlated with forced expiratory volume in 1 s (FEV1) [6,7]. The poorer the lung function, the higher the level of inflammation and the poorer the immune function, such as IL-6, CD8^+^ [8]. The current routine treatment of COPD includes drug therapy, smoking cessation counseling, pulmonary rehabilitation therapy [9], and nutritional supplement, which have gradually entered the clinical treatment [10,11].

Micronutrient intake is inadequate in patients with COPD. It was shown that the intake of calcium, potassium, folate, retinol, and thiamine was lower than recommended dietary allowance (RDA) in over 75% of COPD patients [12]. The intake of vitamins A, C, D, E, B12, carotenoids, and magnesium all have protective effects on the loss of lung function, such as improving the average level of lung function and reducing the rate of decline of lung function indicators [13]. Meanwhile, patients with decreased immune function are susceptible to bacterial and viral infections, leading to worsening clinical symptoms and an increase in the frequency of acute exacerbations, and multiple nutrients play a critical role in regulating immune function and anti-inflammatory effects, mainly reflected in regulating barrier function, inflammatory factors [14].

Although there have been few meta-analyses showing the improvement of vitamin C and D in patients with COPD [15,16], a comprehensive analysis of micronutrients and complex nutrients are currently lacking in COPD patients. Therefore, we conducted this systematic review and meta-analysis to prove the effects of micronutrients on lung function, T-cell immunity, and other indicators of COPD patients.

## 2. Materials and Methods

### 2.1. Search Methods

The PubMed (https://pubmed.ncbi.nlm.nih.gov/), Cochrane Library (https://www.cochranelibrary.com/), and Web of Science databases (https://clarivate.com.cn/solutions/web-of-science/) were searched from their corresponding inception until 12 February 2024. Searching was conducted using keywords including ‘chronic obstructive pulmonary disease’, ‘COPD’, ‘micronutrient’, ‘Dietary Supplements’, ‘vitamin’, ‘mineral’, and ‘randomized controlled trial’. Detailed search strategies are reported in Supplementary Table S1. In addition, the reference lists of critical articles and related meta-analysis articles were also searched in this study. Articles are language-restricted by Chinese and English. This study was registered in the PROSPERO (registration number ID: CRD42023461552).

### 2.2. Inclusion Criteria and Exclusion Criteria

The inclusion criteria were as follows: (1) the subjects were patients diagnosed with COPD and were 18 years old or older in the eligible studies; (2) the study type was randomized controlled trials (RCTs); (3) the studies involved dietary supplementation of various micronutrients (including vitamins and minerals) in the effects on symptoms or outcomes in patients with COPD. Exclusion criteria were: (1) the studies were conducted in cell assays or animal experiments; (2) observational studies: cross-sectional studies, cohort studies, and case-control studies; (3) the studies included non-micronutrients intervention and non-dietary nutrition intervention; (4) the studies included unavailable available data; (5) narrative reviews, conference abstracts, case series, and studies not published in peer-reviewed journals.

### 2.3. Study Selection and Data Extraction

Study selection and data extraction were carried out independently by two different reviewers (ML, LZ). When disagreements arose, a consensus was reached through discussion or reevaluation by the third reviewer (JC). These retrieved articles were imported into EndNote X9.1. After removing duplicate articles, the titles and abstracts of the literature were initially screened according to the inclusion and exclusion criteria. Then the full text was read and screened again, and the study data was extracted for validity check.

The data extracted from the selected research articles included first author, publication year, country, study type, randomized, blinding, sample size, patient style, the characteristic of experimental and control groups, intervention duration, and outcome indicators including FEV1, the ratio of forced expiratory volume in 1 s and forced vital capacity, 6-min walk distance (6MWD), acute exacerbation, COPD assessment test (CAT) score, maximal expiratory pressure (MEP), maximal inspiratory pressure (MIP), and T-cell immunity level, such as CD3^+^, CD4^+^, CD8^+^, and CD4^+^/CD8^+^. For each trial, the means and standard deviation (SDs) of biomarkers at baseline and endpoint in both the control and intervention groups were extracted, respectively. If the trial did not provide the SDs directly, they were calculated from interquartile or standard error of the mean (SEM) using the equation listed in the Cochrane Handbook.

### 2.4. Quality Assessment

The modified Jadad scale was used to evaluate the quality of randomized clinical trials. The modified Jadad scale consists of four parts: random sequence generation, randomization concealment, blinding, and withdrawal. The studies were scored from 0 to 7, and 1–3 signified low quality, while 4–7 signified high quality [17].

### 2.5. Statistical Analysis

Statistical analysis was conducted using STATA 11.0. Heterogeneity among studies was assessed with I^2^ statistics. The I^2^ represented the proportion of total variation, and the high degrees of heterogeneity was defined based on values of 75% as cut-off points. When heterogeneity was high, a random-effects model was chosen, otherwise a fixed-effect model was used. Data were graphically displayed using forest plots. According to different data extracted, the combination effect indicators were selected: weighted mean difference (WMD), standardized mean difference (SMD), and odds ratio (OR) with 95% confidence intervals (CIs). Subgroup analysis of FEV1% and FEV1/FVC% was performed according to geographical differences in the patients with COPD, the style of patients, supplement dosage and vitamin D supplement forms and duration of intervention. Sensitivity analysis and the Egger regression test were conducted to explore the potential sources of heterogeneity. Two sets of data were used to illustrate the effects of various micronutrients on patients with COPD, including differences from baseline and post-intervention (de), and post-intervention indicators (af). There was a statistically significant difference when *p* < 0.05.

## 3. Results

### 3.1. Study Screening and Results

**Figure 1:**
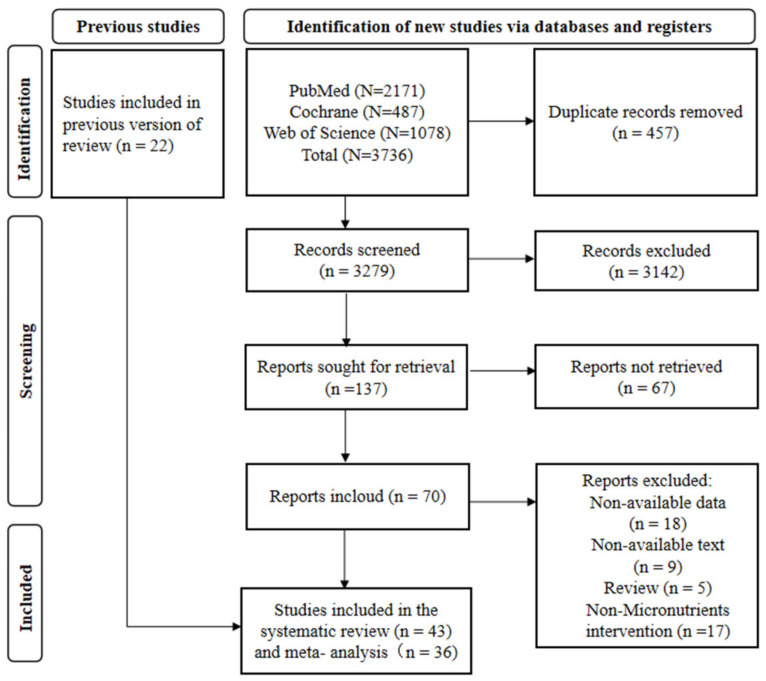
The flow diagram of included and excluded studies.

The literature search following the search strategy returned 3736 potentially relevant records, including 457 duplicate articles. The remaining 3279 papers were screened, and 3142 were preliminarily excluded according to the title and abstract. At the same time, 22 articles were manually retrieved and included in this study. Finally, after reading and analyzing the full text, 43 articles remained in the review for the synthesis of the qualitative analysis, and 36 articles entered the quantitative analysis (Figure 1).

### 3.2. Characteristics of Studies Included

Among all the 43 RCT articles, a total of 4094 participants were included between 1997 and 2022, of which 2225 were in the experiment group and 2069 were in the control group. In all the included studies, 3411 patients were treated alone with vitamin D (1771 in the intervention group and 1640 in the control group), 122 with vitamin C (62 in the intervention group and 60 in the control group), 50 with vitamin E (28 in the intervention group and 22 in the control group), 49 with magnesium (24 in the intervention group and 25 in the control group), and 662 with complex nutrient intervention (340 in the intervention group and 322 in the control group). There were 30 studies from Asia, 11 studies from Europe, and two studies from North America. The main characteristics of the selected studies were shown in Table 1.

### 3.3. Quality Assessment

The quality of each study was evaluated by two independent reviewers. Differences were resolved by the third reviewer. There were 20 articles of high quality and 23 articles of low quality. Specific scores of the included literature were displayed in Supplementary Table S2.

### 3.4. Systematic Review and Meta-Analysis Results

#### 3.4.1. Vitamin D

##### FEV1 and FEV1/FVC%

**Figure 2:**
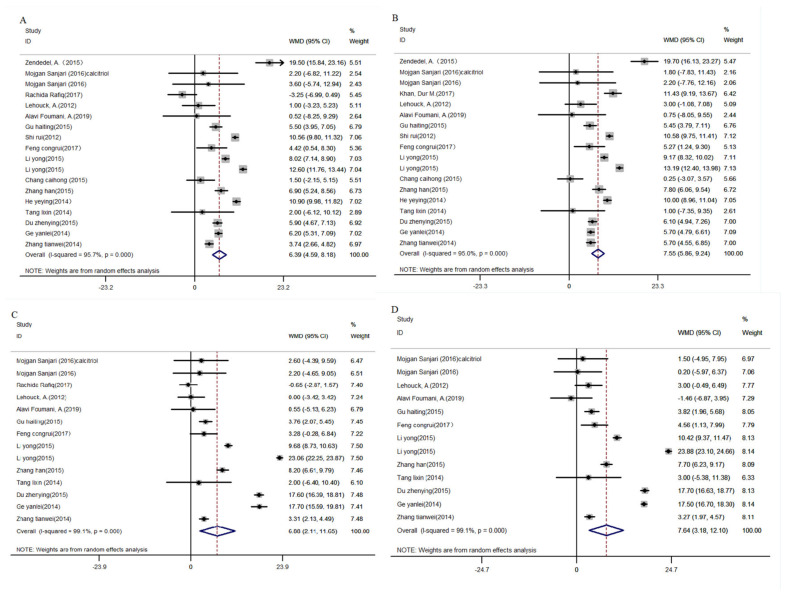
Meta-analysis of vitamin D supplemented in patients with COPD on FEV1 and FEV1/FVC. (**A**): differences of FEV1 between baseline and post-intervention, (**B**): FEV1 of post-intervention, (**C**): differences of FEV1/FVC between baseline and post-intervention, (**D**): FEV1/FVC of post-intervention. FEV1, forced expiratory volume in 1 s; FEV1/FVC, the ratio of forced expiratory volume in 1 s and forced vital capacity; COPD, chronic obstructive pulmonary disease [18,19,20,23,24,27,29,30,31,32,33,34,37,38,39,40,41,42,43].

Nineteen (19) papers that reported the effects of vitamin D supplementation on FEV1 in the experimental group were collected [18,19,20,23,24,27,29,30,31,32,33,34,37,38,39,40,41,42,43]. It was revealed that the level of FEV1 in patients treated with vitamin D supplementation was significantly higher than in patients who did not receive vitamin D supplementation (WMD_de_: 6.39, 95% CI: 4.59, 8.18, *p* < 0.01; WMD_af_: 7.55, 95% CI: 5.86, 9.24, *p* < 0.01, Figure 2A,B). FEV1/FVC% was significantly improved compared to the control group in 11 papers (WMD_de_: 6.88, 95% CI: 2.11, 11.65, *p* < 0.01; WMD_af_: 7.64, 95%CI: 3.18, 12.10, *p* < 0.01, Figure 2C,D) [20,23,27,29,31,32,34,39,40,41,42].

Subgroup analyses indicated decreases in heterogeneity after divided by regional, patient type, duration of intervention, vitamin D supplement form, dose, and literature quality. Vitamin D intervention in Chinese COPD patients with COPD significantly increased lung function (FEV1: WMD_de_: 6.97, 95% CI: 5.12, 8.82, *p* < 0.001; WMD_af_: 7.21, 95% CI: 5.38, 9.05, *p* < 0.001; FEV1/FVC: WMD_de_: 10.05, 95% CI: 4.42, 15.68, *p* < 0.001; WMD_af_: 10.44, 95% CI: 5.29, 15.59, *p* < 0.001), stable patients lung function was significantly increased (FEV1: WMD_de_: 3.09, 95% CI: 1.83, 4.35, *p* < 0.01; WMD_af_: 8.70, 95% CI: 6.31, 11.09, *p* < 0.01; FEV1/FVC: WMD_de_: 9.61, 95% CI: 1.30, 17.92, *p* < 0.01; WMD_af_: 9.83, 95% CI: 0.98, 18.69, *p* < 0.01), and the effect of continuous supplementation was significantly enhanced (FEV1: WMD_de_: 6.40, 95% CI: 4.39, 8.42, *p* < 0.05; WMD_af_: 8.04, 95% CI: 6.32, 9.76, *p* < 0.05; FEV1/FVC: WMD_de_: 7.04, 95% CI: 1.60, 12.48, *p* < 0.05; WMD_af_: 7.84, 95% CI: 2.17, 13.57, *p* < 0.05). The specific results of subgroup analyses were performed in Table 2.

Sensitivity analysis indicated that any included study had no significant impact on the efficacy of combination therapy (Supplementary Figure S1). According to Egger’s regression test, no significant publication bias was found in eligible studies (Egger regression test: *p* > 0.05, Supplementary Figure S2).

##### Other Indicators Related to Lung Function and Disease Severity of COPD

Three studies compared 6MWD after vitamin D supplementation in experimental and control groups [20,25,33]. Overall, it was revealed that there was no significant difference between the two groups according to the random effects model (WMD_de_: 3.15, 95% CI: −22.44, 31.72, *p* > 0.05; WMD_af_: −2.59, 95% CI: −34.16, 28.98, *p* > 0.05, Supplementary Figure S3A,B).

The effect of vitamin D supplementation on acute exacerbations was mentioned in seven articles [18,20,24,27,29,31,38]. The number of acute exacerbations in the supplementary group was found to be less than the control group (OR: 0.36, 95% CI: 0.24, 0.54, *p* < 0.05, Supplementary Figure S3C).

The effect of vitamin D supplementation on muscle strength was mentioned in three articles [20,25,44]. In the literature, two papers [20,25] were included involved the indicators of the strength of the respiratory muscles for meta-analysis. After the intervention of vitamin D, the results demonstrated no heterogeneity of MEP (WMD_de_: 0.25, 95% CI: −0.16, 0.66, *p* > 0.05) and greater consistency of MIP (WMD_de_: −0.30, 95% CI: −1.51, 0.92, *p* > 0.05, Supplementary Figure S3D,E).

The COPD assessment test (CAT) score is an indicator to evaluate the severity of COPD, which was involved in four articles [27,28,36,37]. After vitamin D intervention, the CAT score of the intervention group was significantly lower than that of the control group (WMD_de_: −5.76, 95% CI: −7.76, −3.31, *p* < 0.001; WMD_af_: −5.25, 95% CI: −5.82, −4.69, *p* < 0.001, Supplementary Figure S3F,G).

##### T Cells Level

**Figure 3:**
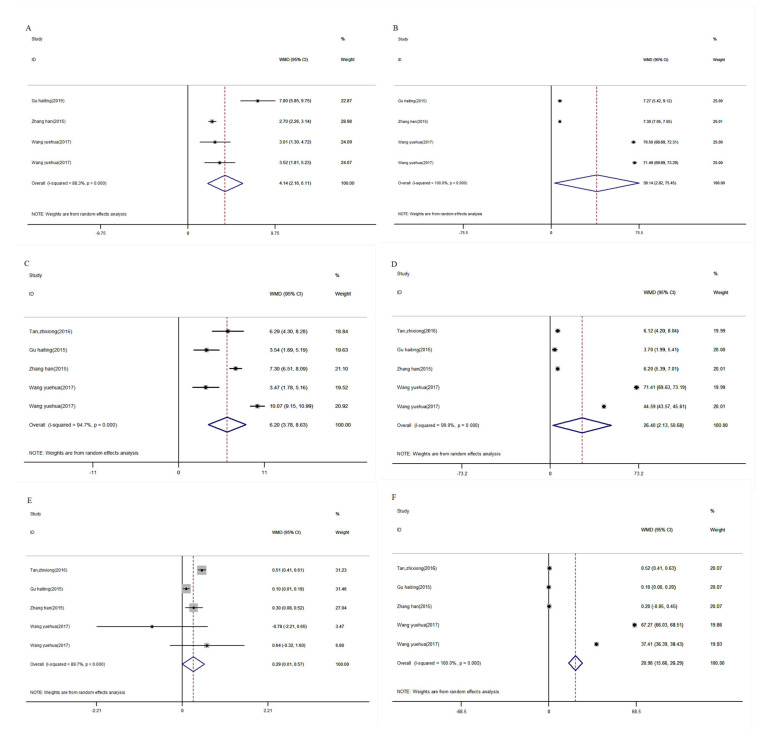
Meta-analysis of vitamin D supplemented in patients with COPD on CD3^+^, CD4^+^, CD4^+^/CD8^+^. (**A**): differences of CD3^+^ between baseline and post-intervention, (**B**): CD3^+^ of post-intervention, (**C**): differences of CD4^+^ between baseline and post-intervention, (**D**): CD4^+^ of post-intervention, (**E**): differences of CD4^+^/CD8^+^ between baseline and post-intervention, (**F**): CD4^+^/CD8^+^ of post-intervention, COPD, chronic obstructive pulmonary disease. [28,29,34,35].

The levels of CD3^+^ [29,34,35] were shown in the Figure 3A,B. The results proved that after vitamin D supplementation, CD3^+^ in the intervention group was significantly higher than that in the control group (WMD_de_: 4.14, 95% CI: 2.16, 6.11, *p* < 0.001; WMD_af_: 39.14, 95% CI: 2.82, 75.45, *p* < 0.001).

Five studies from four papers compared the levels of CD4^+^, CD8^+^, and CD4^+^/CD8^+^ after vitamin D supplementation in experimental and control groups [28,29,34,35]. It was shown that CD4^+^ was significantly higher in the intervention group according to the random effects model (WMD_de_: 6.20, 95% CI: 3.78, 8.63, *p* < 0.001; WMD_af_: 26.40, 95% CI: 2.13, 50.68, *p* < 0.001, Figure 3C,D). Also, a significant improvement in CD4^+^/CD8^+^ was found via vitamin D treatment (WMD_de_: 0.29, 95% CI: 0.01, 0.57, *p* < 0.05, WMD_af_: 20.98, 95% CI: 15.66, 26.29, *p* < 0.05, Figure 3E,F). However, the levels of CD8^+^ suggested no significant differences between the two groups (WMD_de_: −1.80, 95% CI: −4.07, 0.48, *p* > 0.05; WMD_af_: −1.36, 95% CI: −3.43, 0.72, *p* > 0.05, Supplementary Figure S4). 

#### 3.4.2. Vitamin C

Only three articles involved vitamin C supplement alone for COPD patients [42,43,44]. The results of FEV1 stated no significant difference in the comparison of difference data and the data after intervention (WMD_de_: 3.08, 95% CI: −0.64, 6.80, *p* > 0.05; WMD_af_: 1.46, 95% CI: −6.39, 9.31, *p* > 0.05, Supplementary Figure S5A,B) [45,47]. Moreover, the results of FEV1/FVC received the similar results (WMD_de_: 2.83, 95% CI: −1.62, 7.27, *p* > 0.05; WMD_af_: 1.73, 95% CI: −4.83, 8.30, *p* > 0.05, Supplementary Figure S5C,D) [45,46].

#### 3.4.3. Vitamin E

There are few articles on vitamin E supplementation alone, and FEV1% and FEV1/FVC% were meta-analyzed only [45,48]. The results manifested no significant improvement in FEV1% and FEV1/FVC% by vitamin E treatment. The results of FEV1% are shown in Supplementary Figure S6A,B (WMD_de_: −0.30, 95% CI: −6.62, 6.02, *p* > 0.05; WMD_af_: −2.15, 95% CI: −8.60, 4.31, *p* > 0.05). The results of FEV1/FVC% are represented in Supplementary Figure S6C,D (WMD_de_: −3.00, 95% CI: −8.17, 2.17, *p* > 0.05; WMD_af_: −0.79, 95% CI: −6.05, 7.63, *p* > 0.05).

#### 3.4.4. Magnesium

There is only one article on how single-mineral supplementation improves lung function in people with COPD [49]. It was revealed that magnesium supplementation alone did not significantly improve lung function, as well as other indicators.

#### 3.4.5. Compound Nutrients

##### FEV1 and FEV1/FVC%

**Figure 4:**
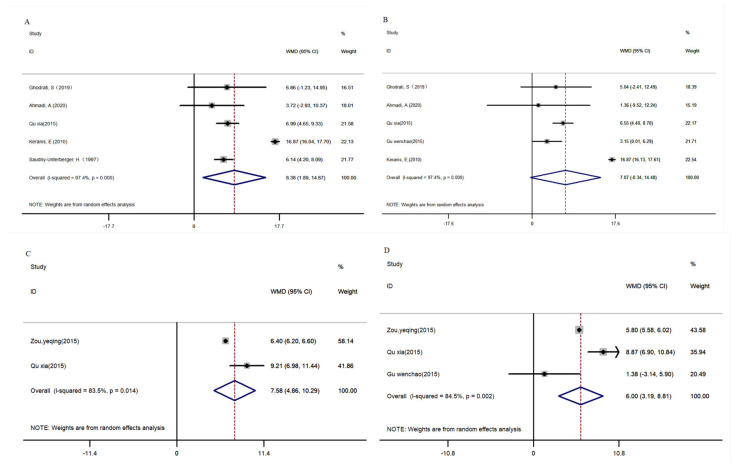
Meta-analysis of compound nutrients supplementation patients with COPD on FEV1 and FEV1/FVC. (**A**): differences of FEV1 between baseline and post-intervention, (**B**): FEV1 of post-intervention, (**C**): differences of FEV1/FVC between baseline and post-intervention, (**D**): FEV1/FVC of post-intervention. FEV1, forced expiratory volume in 1 s, FEV1/FVC, the ratio of forced expiratory volume in 1 s and forced vital capacity, COPD, chronic obstructive pulmonary disease [52,53,54,55,57,58,59,60].

Eight studies were found involving the effects of complex nutritional supplements on lung function in patients, but the number of meta-analysis results was inconsistent due to data issues [52,53,54,55,57,58,59,60], and only seven of these articles were analyzed. The results proved that FEV1 was significantly increased in COPD patients after receiving multinutrient supplementation (WMD_de_: 8.38, 95% CI: 1.89, 14.87, *p* < 0.05; WMD_af_: 7.07, 95% CI: −0.34, 14.48, *p* < 0.05, Figure 4A,B).

In total, three articles illustrated the effects of complex nutrition supplementation on FEV1/FVC [57,59,60]. The results revealed that FEV1/FVC in COPD patients with compound nutrients supplementation increased significantly when compared to the control group (WMD_de_: 7.58, 95% CI: 4.86, 10.29, *p* < 0.05; WMD_af_: 6.00, 95% CI: 3.19, 8.81, *p* < 0.05, Figure 4C,D). 

##### Other Indicators Related to Lung Function and Disease Severity of COPD

The results by meta-analyzing three articles [50,56,60] indicated no significant difference on 6WMD (WMD_de_: −1.87, 95% CI: −5.78, 2.03, *p* > 0.05; WMD_af_: 8.98, 95% CI: 1.53, 16.44, *p* > 0.05, Supplementary Figure S7A,B). Additionally, there was no significant difference on SGRQ by summarizing two articles [51,54] (WMD_de_: −6.08, 95% CI: −14.24, 2.07, *p* > 0.05, WMD_af_: −2.15, 95% CI: −7.97, 3.68, *p* > 0.05, Supplementary Figure S7C,D).

## 4. Discussion

This study analyzed the effects of micronutrients alone or in a compound on lung function in COPD patients through systematic review and meta-analysis. The beneficial effects of vitamin D alone and complex nutrients supplemented in patients were reflected in improving lung function and related disease severity indicators, such as the levels of T cells. In addition, the supplementation of vitamins C and E alone did not have a significant promoting effect on lung function. The improvement in patients supplemented with magnesium may be reflected in reducing the patient’s level of inflammation, while other mineral interventions alone in COPD patients have been sparsely described.

This meta-analysis showed that supplementation of vitamin D alone had a certain improvement effect on lung function and immunity in COPD patients, such as FEV1 and FEV1/FVC, reduced the number of acute exacerbations, and improved the levels of T cells. It was indicated that vitamin D can be given in the general treatment of patients with COPD. The effects of vitamin D supplementation on the lung function were consistent with most of the results of a meta-analysis written by Li et al. [16], but the 6WMD suggested different findings. This may be because the previous meta-analysis did not divide into vitamin D alone or in a complex with other nutrients. The ameliorating effect of vitamin D on COPD might be due to the regulation of the inflammatory state and immunity system in vivo [58]. Vitamin D relieves airway inflammation and can also improve lung function by promoting cell multiplication and reducing cell apoptosis [59,60]. Vitamin D can protect the innate immunity and adaptive immune system in the epithelial mucosa, which is reflected in maintaining the integrity of the epithelial mucosa, inhibiting nuclear factor (NF)-κB in epithelial cells, and reducing the expression of cytokines [61].

There was considerable heterogeneity in the results of vitamin D on lung function index (FEV1%, FEV1/FVC%) in the included literature. Sensitivity analyses and publication bias analyses were performed, and it was demonstrated that no single article affected the results. Subsequently, multi-faceted subgroup analyses were conducted. Although the heterogeneity was reduced in all subgroups, the reduction effect on heterogeneity was not obvious. The reasons were hypothesized as manifold for the high degree of heterogeneity in vitamin D interventions to improve the COPD patients’ lung function. Of course, intervention studies with a larger population and more clear experimental design are still needed to definitively prove this.

Vitamin C and vitamin E are the primary antioxidant nutrients [62]. Oxidative stress is the main driving mechanism of COPD pathogenesis; oxidative stress in the lungs comes from exogenous oxidative stress caused by exposure to smoking or air pollution in and around the city, as well as endogenous oxidative stress generated by activated inflammatory cells, especially neutrophils [63,64]. Therefore, treating oxidative stress with antioxidants or enhancing endogenous antioxidants should be an effective strategy to treat the underlying pathogenesis of COPD [65]. Previous studies have proven that dietary vitamin C supplementation might prevent COPD, and vitamin E intake was positively correlated with FEV1 [66,67]. However, after meta-analysis, we found no significant improvement in vitamin C intervention because there were fewer studies of vitamin C interventions alone. Supplementation with a single antioxidant vitamin has been less studied, possibly because of the limited effectiveness of supplementation, and the combination of antioxidant vitamins and other nutrients have been opted to improve COPD.

It was demonstrated in this study that multinutrient supplementation improved FEV1 and FEV1/FVC in COPD patients but did not significantly effect 6WMD and SGRQ. The many micronutrients contained in the complex nutrients such as vitamins (A, C, D, and E), minerals (zinc, selenium, iron, and magnesium), and a range of others all play important roles in reducing the risk of chronic lung disease and viral infections [68,69,70,71]. Compared with single micronutrients, multiple nutrients are added to supplements to have a stable effect, comprehensive nutrients, and nutrients can also promote mutual absorption, such as vitamin C and vitamin E work together to exert antioxidant effects, and Gu Wenchao also uses vitamin D and calcium in combination to improve COPD patients [51,52,54]. The effect of complex nutrient intervention on COPD may be multifaceted [72]. Comprehensive nutrients can improve the nutritional level of patients, and the nutritional level of patients is also significantly correlated with lung function and quality of life [73,74]. In addition, compound nutrients can improve body composition, and reduce inflammation levels [48,49,56]. The results of our study illustrated that the intervention effect of complex nutrients is mainly focused on improving lung function, and it may be that the addition of vitamin D plays a very important role in lung function. Complex nutrients also have some effect on reducing inflammation levels, possible because the added nutrients exert some anti-inflammatory effects. Therefore, the supplementation of complex nutrients may be a better intervention for the stability of disease status and lung function in patients with COPD.

There were some certain advantages in this systematic review and meta-analysis. So far, this is a full-scale meta-analysis to systematically evaluate the clinical efficacy of micronutrients alone or in a compound supplement, including lung functions and disease severity, in COPD patients with comprehensive consideration. A comprehensive range of micronutrients were included, including supplementation of vitamin D, vitamin C, vitamin E, and magnesium alone, and complex micronutrients. In addition, the variables analyzed in this study were not just post-intervention differences between groups, but also included comparisons of pre- and post-intervention differences, which better eliminated the effects of baseline data. However, few limitations still existed. There were high heterogeneities in a few outcomes, and the sources of heterogeneity were discussed through subgroup analyses, sensitivity analyses, and publication bias. In addition, some of the included literature reviews were of low quality, suggesting that future clinical trial designs should be more rigorous to improve the quality of the literature. We were also able to minimize bias throughout the process by creating detailed protocols, independently selecting articles, and using statistical analysis and data selection.

## 5. Conclusions

In summary, the results found that micronutrient dietary supplementation has important clinical implications for COPD patients. Vitamin D supplementation alone and multinutrient supplementation resulted in significant improvements in lung function and disease severity in patients with COPD. Due to the small number of articles on vitamin C and vitamin E supplementation alone in people with COPD, our results showed no significant effect on improving lung function and need to be further confirmed by large and well-designed prospective randomized controlled trials. Consequently, it is recommended that patients with COPD can properly supplement vitamin D or complex nutrients on the basis of basic treatment.

## Figures and Tables

**Table 1:** Characteristics of included studies.

Author (Year)	Country	Blinding	Sample Size (I, C)	Sex	Age (y)	Patient Style	Intervention Group		Control Group	Duration	Outcome	Jadad Score
							Dosage	Route	Composition (Dosage)			
Vitamin D												
Zendedel, A. (2015) [18]	Iran	DB	88 (44, 44)	M (60) F (28)	-	Severe and very severe COPD	Vitamin D (100,000 IU/m)	Oral	Placebo	6 m	FEV1 (%), number of exacerbations	4
Mojgan Sanjari (2016) [19]	Iran	DB	135 (I_C_: 39, I_V_: 39, 42)	M F	C: 58.4 ± 9.5 Vitamin D: 55.8 ± 9.5 Calcitriol: 55.6 ± 10.4	Moderate to severe COPD and exacerbations	Calcitriol (0.25 μg/d) Vitamin D (50,000 IU/d)	Oral	Placebo (Similar to active drug)	7 d	FEV1%	6
Rachida Rafiq (2017) [20]	Netherland	-	50 (24, 26)	M (26) F (24)	I: 61 ± 5.92 C: 64 ± 3.7	-	Vitamin D (1200 IU/d)	Oral	Placebo	6 m	FEV1%, FEV1/FVC (%), number of exacerbations, 6MWD, MIP, MEP	6
Rachida Rafiq (2022) [21]	Netherland	DB	155 (74, 81)	M F	I: 67 ± 9 C: 65 ± 9	-	Vitamin D3 (16,800 IU/W)	Oral	Placebo	1 y	Exacerbation rate in 1 y	7
Martineau, A.R. (2015) [22]	UK	DB	240 (122, 118)	M F	I: 64.8 ± 7.9 C: 64.5 ± 9.2	-	Vigantol oil with vitamin D3 (120,000 IU/2 m)	Oral	Placebo (Miglyol oil 6 mL)	1 y (six 2-monthly)	Time to first moderate or severe COPD exacerbation	7
Lehouck, A. (2012) [23]	Belgium	DB	182 (91, 91)	M (145) F (37)	I: 68 ± 9 C: 68 ± 8	Moderate to very severe COPD	Vitamin D (100,000 IU/m)	Oral	Placebo (arachidis oleum 4 mL)	1 y	FEV1%, FEV1/FVC	6
Khan, Dur M. (2017) [24]	Pakistan	-	120 (60, 60)	M (78) F (42)	46.28 ± 8.83	-	Vitamin D (2000 IU/d)	Oral	-	6 m	FEV1%, number of exacerbations	2
Hornikx, M. (2012) [25]	Belgium	DB	50 (25, 25)	M (38) F (12)	I: 67 ± 8 C: 69 ± 6	-	Vitamin D (100,000 IU/m)	Oral	Placebo (arachidis oleum 4 mL)	1 y	MIP, MEP, 6MWD	5
Bjerk, S. M. (2013) [26]	USA	_	36 (18, 18)	M	I: 68 ± 8 C: 67.6 ± 7	-	Cholecalciferol (2000 IU/d)	Oral	Placebo	6 w	SGRQ	3
Alavi Foumani, A.(2019) [27]	Iran	DB	63 (32, 31)	M (60) F (3)	I: 67.9 ± 7.9 C: 68.4 ± 7.8	-	Vitamin D3 (50,000 IU/w)	Oral	Placebo	6 m	FEV1%, FEV1/FVC%, number of exacerbations, CAT score	6
Tan, zhixiong (2016) [28]	China	-	106 (53, 53)	M (61) F (55)	I: 53.9 ± 7.8 C: 54.3 ± 8.6	-	Vitamin D3 (100,000 U/d)	intramuscular injection	Blank	2 w	CD4^+^%, CD8^+^%, CD4^+^/CD4^+^%, CAT score	3
Gu haiting (2015) [29]	China	-	172 (86, 86)	M (101) F (71)	I: 65.95 ± 7.56 C: 66.1 ± 7.62	Stable COPD	Alfa-calciferol (0.25 μg/d)	Oral	Blank	6 m	CD3^+^%, CD4^+^%, CD8^+^%, CD4^+^/CD8^+^%, FEV1%, FEV1/FVC%	4
Shi rui (2012) [30]	China	-	72 (36, 36)	M	65.23 ± 11.6	Stable severe COPD	Alfa-calciferol (0.5 μg/d)	Oral	Blank	3 m	FEV1%	3
Li yong (2016) [31]	China	-	150 (I_A_:50, I_B_:50, 50)	M (84) F (66)	I_A_: 65.72 ± 4.98 I_B_: 65.66 ± 4.92 C: 65.6 ± 4.91	Stable COPD	Alfa-calciferol (A: 400 U/d, B:1000 U/d)	Oral	Placebo (starch)	2 m	FEV1%, FEV1/FVC%	3
Feng congrui (2017) [32]	China	-	80 (Stable COPD: 20, 20) (AECOPD: 20, 20)	Stable COPD:M (29) F (11); AECOPD: M (31) F (9)	Stable COPD: I: 74.33 ± 6.43 C: 76.73 ± 5.92 AECOPD: I: 75.20 ± 5.31 C: 75.80 ± 4.86	Stable COPD, Acute exacerbation	Alfa-calciferol (0. 25 μg/d)	Oral	Blank	4 w	FEV1%, FEV1/FVC%	3
Chang caihong (2015) [33]	China	-	80 (40, 40)	M (57) F (33)	I: 59.3 ± 1.2 C: 56.7 ± 0.8	-	Vitamin D	Oral	Blank	30 d	FEV1%, 6MWD	3
Zhang han (2015) [34]	China	-	120 (60, 60)	M (78) F (42)	I: 71 ± 10 C: 73 ± 9	Stable COPD	Alfa-calciferol 0.5 μg/d	Oral	Blank	6 m	CD3^+^%, CD4^+^%, CD8^+^%, CD4^+^/CD8^+^, FEV1%, FEV1/FVC%	3
Wang yuehua (2017) [35]	China	-	150 (I_A_: 50, I_B_: 50, 50)	M (99) F (51)	I_A_: 69.95 ± 3.05 I_B_: 70.12 ± 1.05 C: 67.77 ± 4.34	Stable COPD	Alfa-calciferol (A:0.25 μg/d B:0.5 μg/d)	Oral	Placebo	1 y	CD3^+^%, CD4^+^%, CD4^+^/CD8^+^%	3
Wu yunping (2015) [36]	China	-	89 (44, 45)	M (52) F (37)	I: 53.6 ± 7.1 C: 54.1 ± 9.3	Stable COPD, acute exacerbation	Vitamin D3 (100,000 U/d)	Intramuscular injection	Blank	2 m	CAT score	4
Ma yinbo (2014) [37]	China	-	292 (146, 146)	M (158) F (134)	48.36 ± 6.0	-	Vitamin D then Calcitriol (300,000 U/d + 0.25 μg/d)	Intramuscular injection	Blank	3 m	FEV1%, CAT score	3
He yeying (2014) [38]	China	-	120 (62, 58)	M (87) F (33)	60.5 ± 5. 5	-	Vitamin D then Calcitriol (300,000 U/d + 0.25 μg/d)	Intramuscular injection	Blank	3 m	FEV1%, number of exacerbations	3
Tang lixin (2014) [39]	China	-	60 (30, 30)	M (38) F (22)	55–90	-	Alfa-calciferol (0.5 μg/d)	Oral	Blank	2 m	FEV1%, FEV1/FVC%	3
Du zhenying (2015) [40]	China	-	58 (29, 29)	M (32) F (26)	I: 60.8 ± 11.9 C: 63.1 ± 12.6	Acute exacerbation episode	Vitamin D (4 g/d)	Oral	Blank	2 m	FEV1%, FEV1/FVC%	4
Ge yanlei (2014) [41]	China	-	130 (68, 62)	-	-	Acute exacerbation episode	Vitamin D (800 U/w)	Oral	Blank	2 m	FEV1%, FEV1/FVC%	3
Zhang tianwei (2014) [42]	China	-	350 (175, 175)	M	I: 66.42 ± 7.20 C: 66.38 ± 7.15	Stable COPD	Calcitriol (0.25 μg/d)	Oral	Blank	3 m	FEV1%, FEV1/FVC%	4
Zhangwei (2015) [43]	China	-	200 (100, 100)	M F	I: 45.3 ± 3.4 C: 45.2 ± 3.2	-	Vitamin D (300,000 U/d) + Calcitriol (0.25 μg/d)	Oral	Blank	3 m	FEV1%	4
Knut Sindre Mølmen (2021) [44]	Norway	DB	78 (34, 44)	M F	C: 67 ± 4 I: 69 ± 5	-	Vitamin D (10,000 IU/day, followed by 2000 IU/day)	Oral	placebo (Cold-pressed olive oil)	12 M	Muscle strength, muscle mass, and endurance performance	6
Vitamin C												
Wu, T. C. (2007) [45]	China	-	35 (9, 8)	M F	C: 65.5 (48,75) I: 68 (47, 89)	Stable COPD	Vitamin C (250 mg/d)	Oral	placebo	12 w	FEV1%, FEV1/FVC%	2
Munawar A, A. (2010) [46]	Pakistan	SB	45 (23, 22)	M	C: 55.33 ± 2.19 I: 53.46 ± 1.94	-	Ascorbic acid (1000 mg/d)	Oral	-	1.5 y	FEV1/FVC%,	3
Chen min (2016) [47]	China	-	60 (30, 30)	M (27) F (33)	I: 71.27 ± 3.32 C: 71.57 ± 2.69	Acute exacerbation episode	Vitamin C (500 mg/d)	Oral	Blank	20 d	FEV1%	2
Vitamin E												
Nadeem, A. (2008) [48]	India	SB	24 (10, 14)	M	C: 54.86 ± 7.13 I: 60.10 ± 1.16	_	Vitamin E (800 IU/d)	Oral	Blank	8 w	FEV1%	4
Wu, T. C. (2007) [45]	China	-	35 (I_200_: 9, I_400:_ 9, 8)	M F	C: 65.5 (48, 75) I_400_: 71 (49, 84) I_200_: 72 (51, 86)	Stable COPD	Vitamin E (200 or 400 mg/d)	Oral	Placebo	12 w	FEV1%, FEV1/FVC%	2
Magnesium												
Zanforlini, B. M. (2022) [49]	Italy	DB	49 (25, 24)	M (38) F (11)	I: 73.0 ± 8.9 C: 72.2 ± 11.0	Moderate–severe stable COPD	Magnesium citrate (300 mg/d)	Oral	Placebo (Maltodextrin, riboflavin, orange flavor, citric acid, sucrose, and sodium bicarbonate)	6 m	FEV1%, FEV1/FVC%, 6MWD, SGRQ	6
Compound nutrients												
Van de Bool, Coby (2017) [50]	Netherland	DB	81 (39, 42)	M (41) F (40)	43–80	-	Oral nutritional supplementation (9.4 g proteins, 28.1 g carbohydrates and 4.1 g fat, was enriched with leucine, n−3 PUFA and vitamin D) 2–3 portions	Oral	Placebo (non-caloric aqueous solution 2–3 portions)	4 m	6MWD	7
Martijn van Beer (2020) [51]	Netherland	DB	81 (39, 42)	M (41) F (40)	C: 62.8 ± 1.3 I: 62.2 ± 1.3	-	Oral nutritional supplementation (9.4 g proteins, 28.1 g carbohydrates and 4.1 g fat, was enriched with leucine, n-3 PUFA and vitamin D) (375 mL)	Oral	Placebo (non-caloric aqueous solution) (375 mL)	4 m	SGRQ	7
Saudny-Unterberger, H. (1997) [52]	Canada	-	24 (14, 10)	M (15) F (9)	40–85	-	Oral nutritional support	Oral	-	2 w	FEV1%	3
Ghodrati, S (2019) [53]	Iran	-	40 (20, 20)	M F	I: 62.05 ± 13.58 C: 54.25 ±14.34	Vitamin D deficiency	Calcium-vitamin D (one calcium-vitamin D tablet/d+ vitamin D3 50,000 IU/w)	Oral	Placebo	3 m	FEV1%	2
Ahmadi, A. (2020) [54]	Iran	SB	44 (23, 21)	M	C: 63.47 ± 7.24 I: 62.08 ± 7.0	-	Whey beverage magnesium and vitamin C (each 250 mL contained 275 mg elemental magnesium, 685 mg vitamin C, and 15.9 g whey protein)	Oral	Blank	8 w	FEV1%, SGRQ	5
Keranis, E. (2010) [55]	Greece	-	120 (60, 60)	M (105) F (15)	68.1 ± 1.4	-	Fruit and vegetables	oral	blank	3 y	FEV1%	3
Gouzi, F (2019) [56]	France	-	57 (31, 26)	M (28) F (29)	C: 61.1 ± 8.7 I: 62 4 ± 6.5	Stable COPD	Antioxidant supplementation (α-tocopherol: 30 mg/day, ascorbate: 180 mg/day, zinc gluconate: 15 mg/day, and Selen methionine: 50 μg/day)	Oral	Placebo	4 w	6WMD	4
Zou,yeqing (2015) [57]	China	-	117 (58, 59)	-	-	Stable COPD	Vitamin E (200 mg/d) + vitamin C (300–600 mg/d) + protein	Oral	Blank	6 m	FEV1/FVC%, FEV1%, SGRQ	3
Long,zhuqing (2013) [58]	China	-	45 (25, 20)	M (27) F (18)	I: 45.5 ± 13.2 C: 46.6 ± 3.6	-	Vitamin E(8–10 IU/d) + vitamin C (400–800 IU/d)	Oral	Blank	30 d	FEV1(L)	2
Qu xia (2015) [59]	China	-	74 (37, 37)	M (44) F (30)	49.8	-	Vitamin D (0.25 μg/d) + vitamin A (5000 U/d)	Oral	Blank	3 m	FEV1%, FEV1/FVC%, number of acute exacerbations	3
Gu wenchao (2015) [60]	China	-	60 (30, 30)	M (58) F (12)	I: 65.37 ± 6.23 C: 65.13 ± 7.03	Stable COPD	Puritan’s Pride liquid calcium (1000 U/d) + vitamin D (1200 mg)	Oral	Placebo	12 m	FEV1%, FEV1/FVC%, 6MWD, SGRQ	3

Abbreviations: I: Intervention group, C: control group, M: male, F: female, DB: double-blinding, SB: single-blinding, COPD: chronic obstructive pulmonary disease, AECOPD: acute exacerbations of chronic obstructive pulmonary disease, FEV1: forced expiratory volume in 1 s, FEV1/FVC: the ratio of forced expiratory volume in 1 s and forced vital capacity, 6MWD: 6-min walk distance, MEP: maximal expiratory pressure, MIP: maximal inspiratory pressure, CAT: COPD assessment test, SGRQ: St George’s Respiratory Questionnaire.

**Table 2:** Subgroup analyses of meta-analysis of vitamin D used in patients with COPD on FEV1% and FEV1/FVC.

Subgroup Analyses		FEV1%				FEV1/FVC			
		WMD_(de)_ (95%CI)	*I*^2^(%)	WMD_(af)_ (95%CI)	*I*^2^(%)	WMD_(de)_ (95%CI)	*I*^2^(%)	WMD_(af)_ (95%CI)	*I*^2^(%)
Regional	Chinese	6.39 (4.59, 8.18)	95.7	7.55 (5.86, 9.24)	95.0	6.88 (2.11, 11.65)	99.1	7.64 (3.18, 12.10)	99.1
		6.97 (5.12, 8.82)	96.4	7.21 (5.38, 9.05)	96.1	10.05 (4.42, 15.68)	99.3	10.44 (5.29, 15.59)	99.3
	Other Asian countries	6.91 (−4.47, 12.28)	89.7	8.39 (1.71, 15.06)	87.6	1.61 (−2.10, 5.32)	0.0	−0.10 (−3.54, 3.34)	0.0
	European countries	−1.24 (−5.4, 2.91)	54.0	3.00 (−1.08, 7.08)	-	−0.46 (−2.32, 1.40)	0.0	3.00 (−0.49, 6.49)	-
The style of patients	Stable COPD	3.09 (1.83, 4.35)	98.0	8.70 (6.31, 11,09)	96.8	9.61 (1.30, 17.92)	99.6	9.83 (0.98, 18.69)	99.6
	AECOPD	1.28 (−0.02, 2.57)	96.5	5.81 (5.10, 6.52)	0.0	11.64 (6.60, 16.67)	91.6	11.82 (8.06, 15,58)	94.4
	NA	0.90 (0.12, 1.91)	96.9	7.3 (3.02, 11.58)	92.7	−0.26 (−1.99, 1.47)	0.0	1.83 (−0.93, 4.60)	0.0
	Both	0.71 (0.07, 1.35)	-	5.27 (1.24, 9.30)	-	3.28 (−0.28, 6.84)	-	4.56 (1.13, 7.99)	-
Duration of intervention	≤1 month	2.87 (0.41, 5.33)	0.0	2.35 (−0.46, 5.17)	15.9	2.97 (0.10, 5.85)	0.0	3.17 (0.45, 5.89)	0.0
	2–3 months	7.98 (5.74, 10.22)	97.3	8.3 (6.14, 10.46)	97.0	12.56 (5.56, 19.57)	99.4	13.06 (7.07, 19.05)	99.4
	≥6 months	5.35 (0.60, 10.09)	94.1	8.55 (4.55, 12.56)	92.6	2.59 (−1.17,6.35)	92.1	3.90 (0.64, 7.16)	85.0
Vitamin D supplement form	Vitamin D	5.53 (3.05, 8.00)	94.5	7.04 (4.70, 9.37)	93.9	5.96 (−1.37, 13.29)	98.2	7.58 (2.79, 12.37)	97.3
	Calcitriol	3.72 (2.65, 4.79)	0.0	5.65 (4.50, 6.79)	0.0	3.29 (2.13, 4.45)	0.0	3.20 (1.93, 4.47)	0.0
	Alfa calciferol	8.40 (4.99, 11.82)	95.9	9.21 (6.02, 12.38)	95.5	9.62 (1.33, 17.90)	99.5	10.00 (1.28, 18.71)	99.5
	Vitamin D + Calcitriol	10.90 (9.98, 11.82)	-	10.00 (8.96, 11.04)	-	-	-	-	-
Literature quality	High quality	4.80 (1.91, 7.69)	91.3	6.66 (3.94, 9.38)	88.7	3.79 (−2.10, 9.69)	98.3	4.19 (−2.53, 10.91)	98.5
	Low quality	7.83 (5.92, 9.73)	95.3	8.17 (6.22, 10.13)	95.6	11.00 (4.06, 17.93)	99.2	11.62 (5.71, 17.53)	99.2
Frequency of supplementation	One-time high-dose or spaced supplementation	7.20 (−0.34, 14.74)	94.7	7.68 (0.14, 15.12)	95.0	6.21 (−7.23, 19.64)	97.8	6.53 (−6.20, 19.25)	98.1
	Continuous supplementation	6.40 (4.39, 8.42)	96.0	8.04 (6.32, 9.76)	94.3	7.04 (1.60, 12.48)	99.2	7.84 (2.17, 13.57)	99.3
	NA	1.50 (−2.15, 5.15)	-	0.25 (−0.37, 3.57)	-	-	-	-	-
Dose	<10 μg/d	6.26 (4.33, 8.20)	93.9	7.09 (5.22, 8.96)	90.1	6.79 (3.34, 10.24)	96.3	6.76 (2.04, 11.49)	98.6
	10 μg–100 μg/d	7.55 (−1.02, 16.12)	97.2	10.61 (5.99, 15.23)	97.9	7.51 (−10.96, 25.98)	99.6	13.51 (−6.95, 33.97)	99.2
	>100 μg/d	6.65 (2.42, 10.88)	93.4	6.6 (3.20, 10.16)	89.4	7.06 (−6.03, 20.13)	96.0	5.68 (−9.02, 20.39)	97.3
	NA	1.50 (−2.15, 5.15)	-	0.25 (−0.37, 3.57)	-	-	-	-	-

## Data Availability

The data presented in this study are available on reasonable request from the corresponding author.

## Supplementary Materials

The following supporting information can be downloaded at: https://www.mdpi.com/article/10.3390/nu16071028/s1, Supplementary Table S1: Detailed search strategies. Supplementary Table S2: Jadad score of include study. Supplementary Figure S1: Egger’s regression test for the assessment of publication bias in Meta-analysis of vitamin D supplementation in patients with COPD on FEV1 and FEV1/FVC. Supplementary Figure S2: Sensitivity Analysis in Meta-analysis of vitamin D supplementation in patients with COPD on FEV1 and FEV1/FVC. Supplementary Figure S3: Meta-analysis of vitamin D supplementation in patients with COPD on 6MWD, the number of acute exacerbations, MEP, MIP, and CAT source. Supplementary Figure S4: Meta-analysis of vitamin D supplementation patients with COPD on CD8^+^. Supplementary Figure S5: Meta-analysis of vitamin C supplementation patients with COPD on FEV1 and FEV1/FVC. Supplementary Figure S6: Meta-analysis of vitamin E Supplementation patients with COPD on FEV1 and FEV1/FVC. Supplementary Figure S7: Meta-analysis of compound nutrients supplementation patients with COPD on the score of 6WMD and SGRQ.

## References

[1] GBD 2019 Diseases and Injuries Collaborators (2020). Global burden of 369 diseases and injuries in 204 countries and territories, 1990–2019: A systematic analysis for the Global Burden of Disease Study 2019. Lancet.

[2] Safiri S., Carson-Chahhoud K., Noori M., Nejadghaderi S.A., Sullman M.J.M., Ahmadian Heris J., Ansarin K., Mansournia M.A., Collins G.S., Kolahi A.A. (2022). Burden of chronic obstructive pulmonary disease and its attributable risk factors in 204 countries and territories, 1990–2019: Results from the Global Burden of Disease Study 2019. BMJ Clin. Res. Ed..

[3] Venkatesan P. (2023). GOLD COPD report: 2023 update. Lancet Respir. Med..

[4] Vogelmeier C.F., Román-Rodríguez M., Singh D., Han M.K., Rodríguez-Roisin R., Ferguson G.T. (2020). Goals of COPD treatment: Focus on symptoms and exacerbations. Respir. Med..

[5] Celli B.R., Cote C.G., Marin J.M., Casanova C., Montes de Oca M., Mendez R.A., Pinto Plata V., Cabral H.J. (2004). The body-mass index, airflow obstruction, dyspnea, and exercise capacity index in chronic obstructive pulmonary disease. N. Engl. J. Med..

[6] den Otter I., Willems L.N., van Schadewijk A., van Wijngaarden S., Janssen K., de Jeu R.C., Sont J.K., Sterk P.J., Hiemstra P.S. (2016). Lung function decline in asthma patients with elevated bronchial CD8, CD4 and CD3 cells. Eur. Respir. J..

[7] Nakagiri T., Warnecke G., Avsar M., Thissen S., Kruse B., Kühn C., Ziehme P., Knöfel A.K., Madrahimov N., Okumura M. (2012). Lung function early after lung transplantation is correlated with the frequency of regulatory T cells. Surg. Today.

[8] Zhou J., Jin F., Wu F. (2021). Clinical significance of changes in serum inflammatory factors in patients with chronic obstructive pulmonary disease and pulmonary infection. J. Int. Med. Res..

[9] Labaki W.W., Rosenberg S.R. (2020). Chronic Obstructive Pulmonary Disease. Ann. Intern. Med..

[10] Scoditti E., Massaro M., Garbarino S., Toraldo D.M. (2019). Role of Diet in Chronic Obstructive Pulmonary Disease Prevention and Treatment. Nutrients.

[11] Itoh M., Tsuji T., Nemoto K., Nakamura H., Aoshiba K. (2013). Undernutrition in patients with COPD and its treatment. Nutrients.

[12] Laudisio A., Costanzo L., Di Gioia C., Delussu A.S., Traballesi M., Gemma A., Antonelli Incalzi R. (2016). Dietary intake of elderly outpatients with chronic obstructive pulmonary disease. Arch. Gerontol. Geriatr..

[13] Zhai T., Li S., Hu W., Li D., Leng S. (2018). Potential Micronutrients and Phytochemicals against the Pathogenesis of Chronic Obstructive Pulmonary Disease and Lung Cancer. Nutrients.

[14] Gombart A.F., Pierre A., Maggini S. (2020). A Review of Micronutrients and the Immune System-Working in Harmony to Reduce the Risk of Infection. Nutrients.

[15] Lei T., Lu T., Yu H., Su X., Zhang C., Zhu L., Yang K., Liu J. (2022). Efficacy of Vitamin C Supplementation on Chronic Obstructive Pulmonary Disease (COPD): A Systematic Review and Meta-Analysis. Int. J. Chronic Obstr. Pulm. Dis..

[16] Li X., He J., Yu M., Sun J. (2020). The efficacy of vitamin D therapy for patients with COPD: A meta-analysis of randomized controlled trials. Ann. Palliat. Med..

[17] Jadad A.R., Moore R.A., Carroll D., Jenkinson C., Reynolds D.J., Gavaghan D.J., McQuay H.J. (1996). Assessing the quality of reports of randomized clinical trials: Is blinding necessary?. Control. Clin. Trials.

[18] Zendedel A., Gholami M., Anbari K., Ghanadi K., Bachari E.C., Azargon A. (2015). Effects of Vitamin D Intake on FEV1 and COPD Exacerbation: A Randomized Clinical Trial Study. Glob. J. Health Sci..

[19] Sanjari M., Soltani A., Habibi Khorasani A., Zareinejad M. (2016). The effect of vitamin D on COPD exacerbation: A double blind randomized placebo-controlled parallel clinical trial. J. Diabetes Metab. Disord..

[20] Rafiq R., Prins H.J., Boersma W.G., Daniels J.M., den Heijer M., Lips P., de Jongh R.T. (2017). Effects of daily vitamin D supplementation on respiratory muscle strength and physical performance in vitamin D-deficient COPD patients: A pilot trial. Int. J. Chronic Obstr. Pulm. Dis..

[21] Rafiq R., Aleva F.E., Schrumpf J.A., Daniels J.M., Bet P.M., Boersma W.G., Bresser P., Spanbroek M., Lips P., van den Broek T.J. (2022). Vitamin D supplementation in chronic obstructive pulmonary disease patients with low serum vitamin D: A randomized controlled trial. Am. J. Clin. Nutr..

[22] Martineau A.R., James W.Y., Hooper R.L., Barnes N.C., Jolliffe D.A., Greiller C.L., Islam K., McLaughlin D., Bhowmik A., Timms P.M. (2015). Vitamin D3 supplementation in patients with chronic obstructive pulmonary disease (ViDiCO): A multicentre, double-blind, randomised controlled trial. The Lancet. Respir. Med..

[23] Lehouck A., Mathieu C., Carremans C., Baeke F., Verhaegen J., Van Eldere J., Decallonne B., Bouillon R., Decramer M., Janssens W. (2012). High doses of vitamin D to reduce exacerbations in chronic obstructive pulmonary disease: A randomized trial. Ann. Intern. Med..

[24] Khan D.M., Ullah A., Randhawa F.A., Iqtadar S., Butt N.F., Waheed K. (2017). Role of Vitamin D in reducing number of acute exacerbations in Chronic Obstructive Pulmonary Disease (COPD) patients. Pak. J. Med. Sci..

[25] Hornikx M., Van Remoortel H., Lehouck A., Mathieu C., Maes K., Gayan-Ramirez G., Decramer M., Troosters T., Janssens W. (2012). Vitamin D supplementation during rehabilitation in COPD: A secondary analysis of a randomized trial. Respir. Res..

[26] Bjerk S.M., Edgington B.D., Rector T.S., Kunisaki K.M. (2013). Supplemental vitamin D and physical performance in COPD: A pilot randomized trial. Int. J. Chronic Obstr. Pulm. Dis..

[27] Alavi Foumani A., Mehrdad M., Jafarinezhad A., Nokani K., Jafari A. (2019). Impact of vitamin D on spirometry findings and quality of life in patients with chronic obstructive pulmonary disease: A randomized, double-blinded, placebo-controlled clinical trial. Int. J. Chronic Obstr. Pulm. Dis..

[28] Tan Z.X., Ye Z.T., Fu W.Q. (2016). Effect of vitamin D on immunomodulatory function and quality of life in patients with copd. J. Clin. Pulmonol..

[29] Gu H.T., Shao H.Y., Jing X.H., Mao W.W. (2015). Clinical study of alfacalcidol soft capsule on immune function in patients with chronic obstructive pulmonary disease. Chin. J. Clin. Pharmacol..

[30] Shi R., Huang H., Fang Z.Y., Wang J., Gao B. (2012). Study on impact of vitamin D supplementation on serum 25(OH) D and FEV1 in patients with chronic obstructive pulmonary disease. J. Clin. Exp. Med..

[31] Li Y., Chen Z.L. (2016). Effects of vitamin D on acute exacerbation and mortality in patients with COPD. Mod. Pract. Med..

[32] Feng C.R., He L.M., Xu G., Li B.K. (2017). Effect of vitamin D supplementation on COPD in elderly patients and its effect on serum IL-33 expression in patients. J. Pract. Med..

[33] Chang C.H. (2015). Effects of vitamin D on chronic obstructive pulmonary disease. Chin. Community Physician.

[34] Zhang H., Gong J.H., Zhang J.H., Ma J.P. (2015). The value of vitamin D in the treatment of stable patients with chronic obstructive pulmonary disease. Lab. Med. Clin..

[35] Wang Y.H. (2017). Effects of vitamin D on T lymphocyte subsets and lung function in patients with stable COPD. Zhejiang Med. Educ..

[36] Wu Y.P., Hu Q.M., Liu W., Zhou Z.H. (2013). Effects of vitamin D adjuvant therapy on quality of life in patients with chronic obstructive pulmonary disease. Clin. Meta-Anal..

[37] Ma Y.B. (2014). The adjunctive role of vitamin D in the treatment of COPD. Health All.

[38] He Y.Y., Geng Y.D. (2014). The application value of vitamin D in the adjuvant therapy of chronic obstructive pulmonary disease. J. Pract. Cardiovasc. Cerebrovasc. Dis..

[39] Tang L.X., Zhang Y., Yuan Q.Y. (2014). The application of vitamin D in the treatment of patients with chronic obstructive pulmonary disease. Int. J. Lab. Med..

[40] Du Z.Y. (2015). Efficacy of vitamin D in the treatment of patients with acute exacerbation of chronic obstructive pulmonary disease complicated by hypocalcemia. Mod. Diagn. Treat..

[41] Ge Y.L., Li J., Wang H.Y., Ge X.L. (2014). Effect of vitamin D on hypocalcemia in patients with acute exacerbation of chronic obstructive pulmonary disease. Chin. J. Gerontol..

[42] Zhang T.W., Fu H.W., Mao L.Q., Huang M. (2014). Effects of vitamin D supplementation on bone mineral density and inflammatory cytokines in COPD patients. Clin. Medicat. J..

[43] Zhang W. (2015). The adjunctive role of vitamin D in the treatment of COPD. Health Today.

[44] Molmen K.S., Hammarstrom D., Pedersen K., Lian Lie A.C., Steile R.B., Nygaard H., Khan Y., Hamarsland H., Koll L., Hanestadhaugen M. (2021). Vitamin D(3) supplementation does not enhance the effects of resistance training in older adults. J. Cachexia Sarcopenia Muscle.

[45] Wu T.C., Huang Y.C., Hsu S.Y., Wang Y.C., Yeh S.L. (2007). Vitamin E and Vitamin C Supplementation in Patients with Chronic Obstructive Pulmonary Disease. Int. J. Vitam. Nutr. Res..

[46] Ansari M.A., Ansari S., Memon Z. (2010). Does antioxidant ascorbic acid supplementation delay lung function deterioration in stable patients with chronic obstructive? Pulmonary disease. Rawal Med. J..

[47] Chen M., Jin Z.X., Bi H., Du J.Y. (2016). Research of vitamin C in the treatment of patients with AECOPD. J. Clin. Pulm. Med..

[48] Nadeem A., Raj H.G., Chhabra S.K. (2008). Effect of vitamin E supplementation with standard treatment on oxidant-antioxidant status in chronic obstructive pulmonary disease. Indian J. Med. Res..

[49] Zanforlini B.M., Ceolin C., Trevisan C., Alessi A., Seccia D.M., Noale M., Maggi S., Guarnieri G., Vianello A., Sergi G. (2022). Clinical trial on the effects of oral magnesium supplementation in stable-phase COPD patients. Aging Clin. Exp. Res..

[50] van de Bool C., Rutten E.P.A., van Helvoort A., Franssen F.M.E., Wouters E.F.M., Schols A. (2017). A randomized clinical trial investigating the efficacy of targeted nutrition as adjunct to exercise training in COPD. J. Cachexia Sarcopenia Muscle.

[51] van Beers M., Rutten-van Mölken M.P.M.H., van de Bool C., Boland M., Kremers S.P.J., Franssen F.M.E., van Helvoort A., Gosker H.R., Wouters E.F., Schols A.M.W.J. (2020). Clinical outcome and cost-effectiveness of a 1-year nutritional intervention programme in COPD patients with low muscle mass: The randomized controlled NUTRAIN trial. Clin. Nutr..

[52] Saudny-Unterberger H., Martin J.G., Gray-Donald K. (1997). Impact of nutritional support on functional status during an acute exacerbation of chronic obstructive pulmonary disease. Am. J. Respir. Crit. Care Med..

[53] Ghodrati S., Ezzatpanah A., Asadi-Khiavi M., Alian Samakkah S., Esmaeilzadeh A., Pezeshgi A. (2019). Administration of vitamin D to ameliorate dyspnea of chronic obstructive pulmonary disease patients: A randomized controlled trial. Immunopathol. Persa.

[54] Ahmadi A., Eftekhari M.H., Mazloom Z., Masoompour M., Fararooei M., Eskandari M.H., Mehrabi S., Bedeltavana A., Famouri M., Zare M. (2020). Fortified whey beverage for improving muscle mass in chronic obstructive pulmonary disease: A single-blind, randomized clinical trial. Respir. Res..

[55] Keranis E., Makris D., Rodopoulou P., Martinou H., Papamakarios G., Daniil Z., Zintzaras E., Gourgoulianis K.I. (2010). Impact of dietary shift to higher-antioxidant foods in COPD: A randomised trial. Eur. Respir. J..

[56] Gouzi F., Maury J., Heraud N., Molinari N., Bertet H., Ayoub B., Blaquiere M., Bughin F., De Rigal P., Poulain M. (2019). Additional Effects of Nutritional Antioxidant Supplementation on Peripheral Muscle during Pulmonary Rehabilitation in COPD Patients: A Randomized Controlled Trial. Oxid. Med. Cell Longev..

[57] Zou Y.Q., Zou H.Y., Jiang Y.Q., Lou F.X., Zou T. (2015). Influence of antioxidant vitamins combined with moderate protein diet on lung function and quality of life of COPD patients in stable phase. Chin. Nurs. Res..

[58] Long Z.Q. (2013). Chronic obstructive pulmonary disease patients breathing muscle recovery and vitamin C, E relationship. Natl. Med. Front. China.

[59] Qu X., Han D.S., Li Y.P., He H.X., Li M. (2015). The adjuvant therapeutic effect of vitamin A and vitamin D on stable COPD patients. J. Pract. Med..

[60] Gu W.C., Qi G.S., Yuan Y.P., Yang H., Wu H., Tang Z.J., Wang L.X., Feng H.L., Wang H.Z. (2015). Clinical study on the efficacy of vitamin D in delaying the progression of chronic obstructive pulmonary disease. Chin. Med. J..

[61] Valle M.S., Russo C., Casabona A., Crimi N., Crimi C., Colaianni V., Cioni M., Malaguarnera L. (2022). Anti-inflammatory role of vitamin D in muscle dysfunctions of patients with COPD: A comprehensive review. Minerva Medica.

[62] Białek-Gosk K., Rubinsztajn R., Białek S., Paplińska-Goryca M., Krenke R., Chazan R. (2018). Serum Vitamin D Concentration and Markers of Bone Metabolism in Perimenopausal and Postmenopausal Women with Asthma and COPD. Adv. Exp. Med. Biol..

[63] Zheng S., Yang J., Hu X., Li M., Wang Q., Dancer R.C.A., Parekh D., Gao-Smith F., Thickett D.R., Jin S. (2020). Vitamin D attenuates lung injury via stimulating epithelial repair, reducing epithelial cell apoptosis and inhibits TGF-β induced epithelial to mesenchymal transition. Biochem. Pharmacol..

[64] Schrumpf J.A., van der Does A.M., Hiemstra P.S. (2020). Impact of the Local Inflammatory Environment on Mucosal Vitamin D Metabolism and Signaling in Chronic Inflammatory Lung Diseases. Front. Immunol..

[65] Higgins M.R., Izadi A., Kaviani M. (2020). Antioxidants and Exercise Performance: With a Focus on Vitamin E and C Supplementation. Int. J. Environ. Res. Public Health.

[66] Barnes P.J. (2022). Oxidative Stress in Chronic Obstructive Pulmonary Disease. Antioxidants.

[67] Kirkham P., Rahman I. (2006). Oxidative stress in asthma and COPD: Antioxidants as a therapeutic strategy. Pharmacol. Ther..

[68] Schols A. (2013). Nutrition as a metabolic modulator in COPD. Chest.

[69] Park H.J., Byun M.K., Kim H.J., Kim J.Y., Kim Y.I., Yoo K.H., Chun E.M., Jung J.Y., Lee S.H., Ahn C.M. (2016). Dietary vitamin C intake protects against COPD: The Korea National Health and Nutrition Examination Survey in 2012. Int. J. Chronic Obstr. Pulm. Dis..

[70] Hanson C., Lyden E., Furtado J., Campos H., Sparrow D., Vokonas P., Litonjua A.A. (2016). Serum tocopherol levels and vitamin E intake are associated with lung function in the normative aging study. Clin. Nutr..

[71] Gozzi-Silva S.C., Teixeira F.M.E., Duarte A., Sato M.N., Oliveira L.M. (2021). Immunomodulatory Role of Nutrients: How Can Pulmonary Dysfunctions Improve?. Front. Nutr..

[72] Holford P., Carr A.C., Jovic T.H., Ali S.R., Whitaker I.S., Marik P.E., Smith A.D. (2020). Vitamin C-An Adjunctive Therapy for Respiratory Infection, Sepsis and COVID-19. Nutrients.

[73] Talaei M., Hughes D.A., Mahmoud O., Emmett P.M., Granell R., Guerra S., Shaheen S.O. (2021). Dietary intake of vitamin A, lung function and incident asthma in childhood. Eur. Respir. J..

[74] Tabatabaeizadeh S.A. (2022). Zinc supplementation and COVID-19 mortality: A meta-analysis. Eur. J. Med. Res..

