# Supplementary material for: Improvement of Lung Function by Micronutrient Supplementation in Patients with COPD: A Systematic Review and Meta-Analysis
Source: Nutrients. 2024 Apr 1;16(7):1028. doi: 10.3390/nu16071028 (PMC11013492; doi:10.3390/nu16071028)
Supplement: Supplementary file 1 [file nutrients-16-01028-s001.zip › supplement.pdf]

Supplementary Table S1: Detailed search strategies

Datebase	Search strategy
PUBMED	((bronchitis, chronic[MeSH Terms]) OR (chronic bronchitis[Title/Abstract]) OR (pulmonary disease, chronic obstructive[MeSH Terms]) OR (chronic obstructive pulmonary disease[Title/Abstract]) OR"Lung diseases"[MeSH Terms] OR (lung disease[Title/Abstract]) OR (pulmonary disease[Title/Abstract]) OR (COPD[Title/Abstract])) AND ((clinical trial[Title/Abstract]) OR (Randomised[Title/Abstract]) OR (Randomized[Title/Abstract]) OR (randomized controlled trial[Title/Abstract]) OR (Placebo[Title/Abstract])) AND ((Dietary Supplements[MeSH Terms]) OR (Dietary Supplements[Title/Abstract]) OR (micronutrient[Title/Abstract]) OR (vitamin[Title/Abstract]) OR (mineral[Title/Abstract]) OR (vitamin A[Title/Abstract]) OR (retinol[Title/Abstract]) OR (β-Carotene[Title/Abstract]) OR (vitamin C[Title/Abstract]) OR (ascorbic acid[Title/Abstract]) OR (vitamin E [Title/Abstract]) OR (tocopherol[Title/Abstract]) OR (vitamin D[Title/Abstract]) OR (calciferol[Title/Abstract]) OR (antirachitic vitamin[Title/Abstract]) OR (calcium[Title/Abstract]) OR (iron[Title/Abstract]) OR (zinc[Title/Abstract]) OR (magnesium[Title/Abstract]) OR (selenium[Title/Abstract]))
The Cochrane Library	#1 MeSH descriptor: [Pulmonary Diseases, Chronic Obstructive] explode all trees<br>#2 (Chronic Obstructive Pulmonary Diseases): ti,ab,kw<br>#3 (COPD): ti,ab,kw<br>#4 (clinical trial): ti,ab,kw OR (Randomized): ti,ab,kw OR (randomized controlled trail): ti,ab,kw<br>#5 MeSH descriptor: [Dietary Supplements] explode all trees<br>#6 (Dietary Supplements): ti,ab,kw OR (micronutrients): ti,ab,kw OR (mineral): ti,ab,kw OR (vitamin): ti,ab,kw OR (vitamin A): ti,ab,kw<br>#7 (β-carotene): ti,ab,kw OR (retinol): ti,ab,kw OR (vitamin C): ti,ab,kw OR (ascorbic acid): ti,ab,kw OR (vitamin E): ti,ab,kw OR (tocopherol): ti,ab,kw<br>#8 (vitamin D): ti,ab,kw OR (calciferol): ti,ab,kw OR (antirachitic vitamin): ti,ab,kw<br>#9 (calcium): ti,ab,kw OR (iron): ti,ab,kw OR (zinc): ti,ab,kw OR (magnesium): ti,ab,kw OR (selenium): ti,ab,kw<br>#10 (#1 OR #2 OR #3) AND (#4) AND (#5 OR #6 OR #7 OR #8 OR #9)

Web of Science	((((TS= (chronic obstructive pulmonary disease)) OR (TS= (bronchitis, chronic)) OR (AB=(chronic obstructive pulmonary disease)) OR (TS= (COPD))OR (AB=(COPD)))) AND ((((TS=(clinical trial)) OR (AB=(clinical trial)) OR (AB=(Randomised)) OR (TS=(Randomized)) OR (AB=(Randomized)) OR (TS=(randomized controlled trial)) OR (AB=(Placebo)))) AND (((((TS=(Dietary Supplements)) OR (TS=(micronutrient)) OR (TS=(vitamin)) OR (TS=mineral)) OR (TS=(vitamin A)) OR (TS=(retinol)) OR (TS=β-Carotene)) OR (TS=(vitamin C)) OR (TS=(ascorbic acid)) OR (TS=(vitamin E)) OR (TS=(tocopherol)) OR (TS=vitamin D)) OR (TS=(calciferol)) OR (TS=(antirachitic vitamin)) OR(TS=(calcium)) OR (TS=(iron)) OR (TS=zinc)) OR (TS=(magnesium)) OR (TS=(selenium))))

Supplementary Table S2: Jadad score of include study

Code	Author（year）	The generation of Random Sequences	Randomization and Allocation Concealment	Blinding	Drop out and Withdrawal	Total score
1	Zendedel, A.（2015）	1	1	2	0	4
2	Wu, T. C. (2007)	1	1	0	0	2
3	Van de Bool, Coby (2017)	2	2	2	1	7
4	Martijn van Beers（2020）	2	2	2	1	7
5	Saudny-Unterberger, H.（1997）	1	1	0	1	3
6	Mojgan Sanjari (2016)	1	2	2	1	6
7	Rachida Rafiq (2017)	2	2	1	1	6
8	Rachida Rafiq (2020)	2	2	2	1	7
9	Martineau, A.R. (2015)	2	2	2	1	7
10	Lehouck, A. (2012)	1	2	2	1	6
11	Khan, Dur M. (2017)	1	1	0	0	2
12	Hornikx, M. (2012)	1	1	2	1	5
13	Ghodrati, S（2019）	1	1	0	0	2
14	Bjerk, S. M. (2013)	1	1	0	1	3
15	Alavi Foumani, A. (2019)	1	2	2	1	6
16	Ahmadi, A. (2020)	1	2	1	1	5
17	Nadeem, A. (2008)	2	1	1	0	4
18	Keranis, E. (2010)	1	1	0	1	3

19	Gouzi, F (2019)	1	2	0	1	4
20	Munawar A, A. (2010)	1	1	1	0	3
21	Zou yeqing (2015)	1	1	0	1	3
22	Chen min (2016)	1	1	0	0	2
23	Long zhuqing (2013)	1	1	0	0	2
24	Tan zhixiong (2016)	1	1	0	1	3
25	Gu haiting (2015)	2	1	0	1	4
26	Shi rui (2012)	1	1	0	1	3
27	Feng congrui (2017）	1	1	0	1	3
28	Qu xia (2015)	1	1	0	1	3
29	Li yong (2015)	1	1	0	1	3
30	Chang caihong (2015)	1	1	0	1	3
31	Zhang han (2015)	1	1	0	1	3
32	Wang yuehua (2017)	1	1	0	1	3
33	Wu yunping (2015)	2	1	0	1	4
34	Gu wenchao (2015)	1	1	0	1	3
35	Ma yinbo (2014)	1	1	0	1	3
36	He huaying (2014）	1	1	0	1	3
37	Tang lixin (2014)	1	1	0	1	3
38	Du zhenying (2015)	2	1	0	1	4
39	Ge yanlei (2014)	1	1	0	1	3
40	Zhang tianwei (2014)	2	1	0	1	4
41	Zhang wei (2015)	2	1	0	1	4
42	Knut Sindre Mølmen(2021)	1	2	2	1	6

43	Zanforlini, B. M. (2022)	2	2	2	0	6

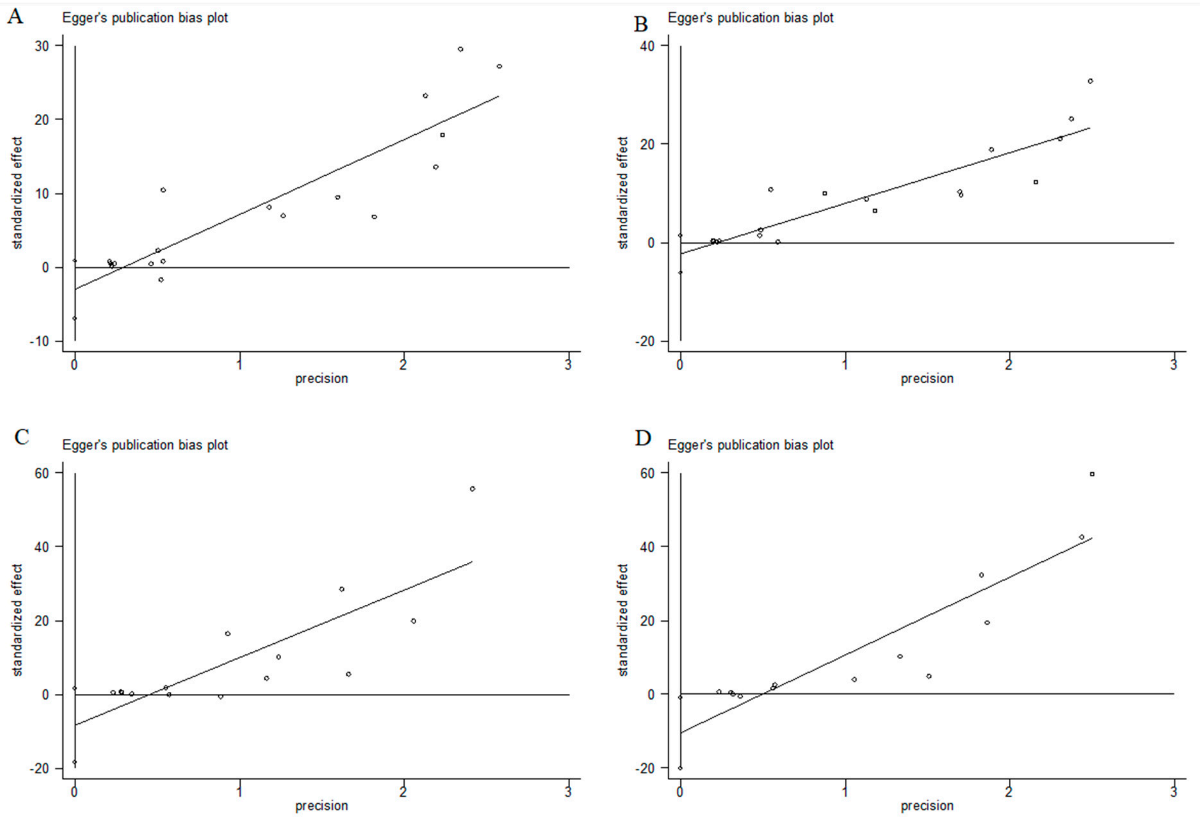

Supplementary Figure S1: Egger's regression test for the assessment of publication bias in Meta-analysis of vitamin D supplementation in patients with COPD on FEV1 and FEV1/FVC. A: differences of FEV1 between baseline and post-intervention, B: FEV1 of post-intervention, C: differences of FEV1/FVC between baseline and post-intervention, D: FEV1/FVC of post-intervention. FEV1, forced expiratory volume in 1 second, FEV1/FVC, the ratio of forced expiratory volume in 1 second and forced vital capacity, COPD, chronic obstructive pulmonary disease.

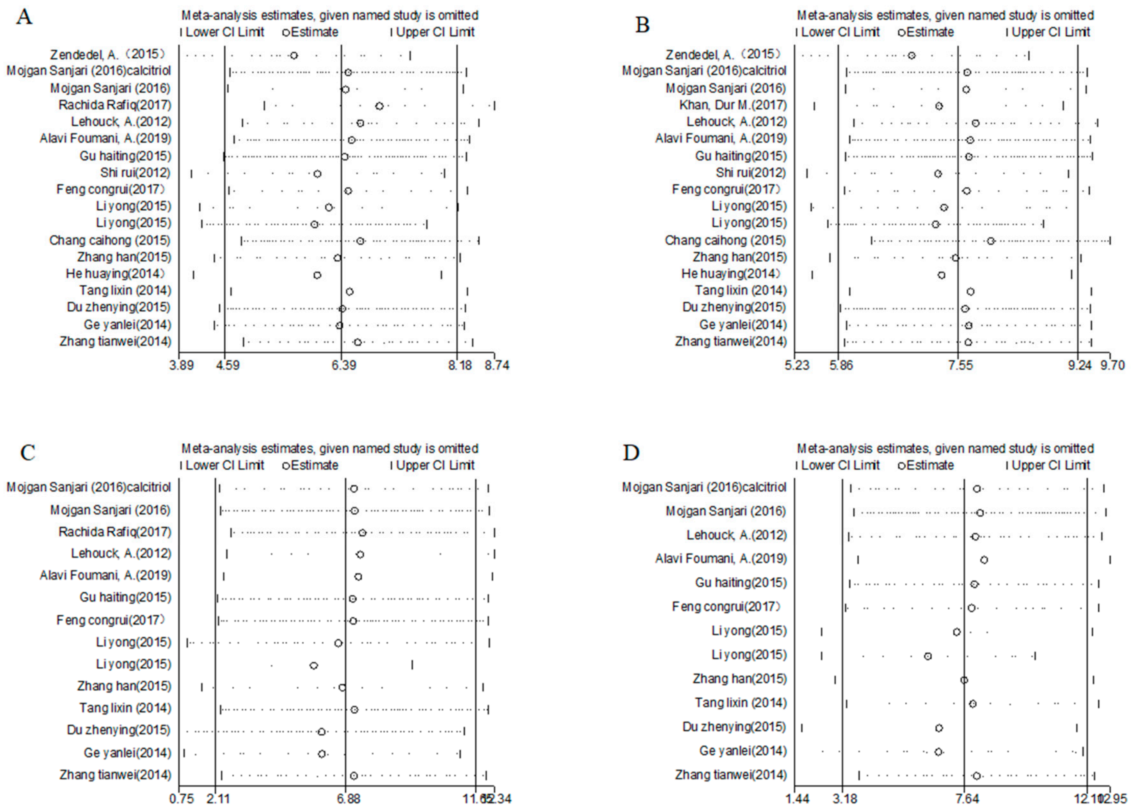

Supplementary Figure S2: Sensitivity Analysis in Meta-analysis of vitamin D supplementation in patients with COPD on FEV1 and FEV1/FVC. A: differences of FEV1 between baseline and post-intervention, B: FEV1 of post-intervention, C: differences of FEV1/FVC between baseline and post-intervention, D: FEV1/FVC of post-intervention. FEV1, forced expiratory volume in 1 second, FEV1/FVC, the ratio of forced expiratory volume in 1 second and forced vital capacity, COPD, chronic obstructive pulmonary disease.

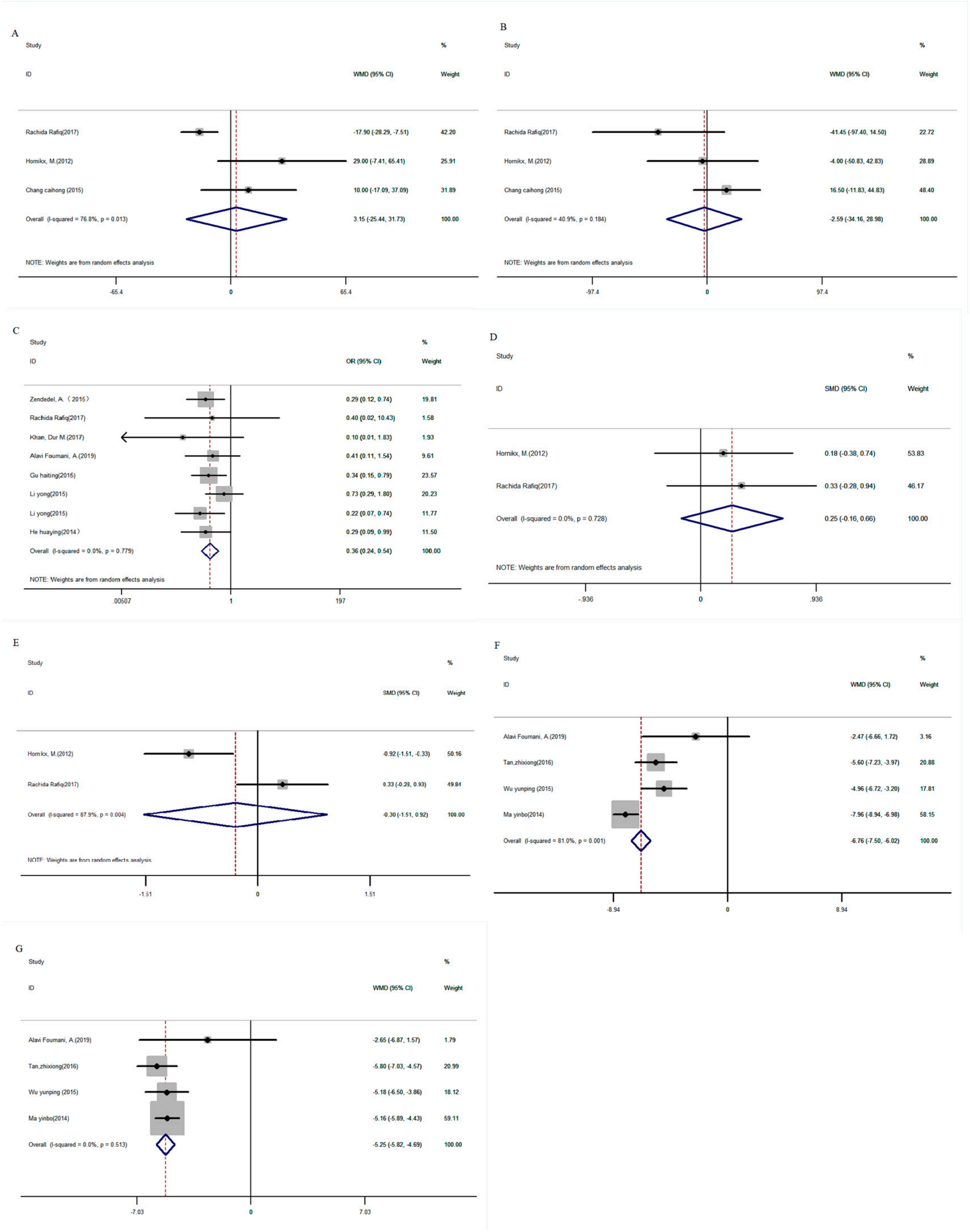

Supplementary Figure S3: Meta-analysis of vitamin D supplementation in patients with COPD on 6MWD, the number of acute exacerbations, MEP, MIP, and CAT source. A: differences of 6MWD between baseline and post-intervention, B: 6MWD of post-intervention, C: the number of acute exacerbations, D: MEP of post-intervention, E: MIP of post-intervention, F: differences of CAT between baseline and post-intervention, G: CAT of post-intervention. 6MWD, 6-minute walk distance, MEP, maximal expiratory pressure, MIP, maximal inspiratory pressure, CAT, COPD assessment test, COPD, chronic obstructive pulmonary disease.

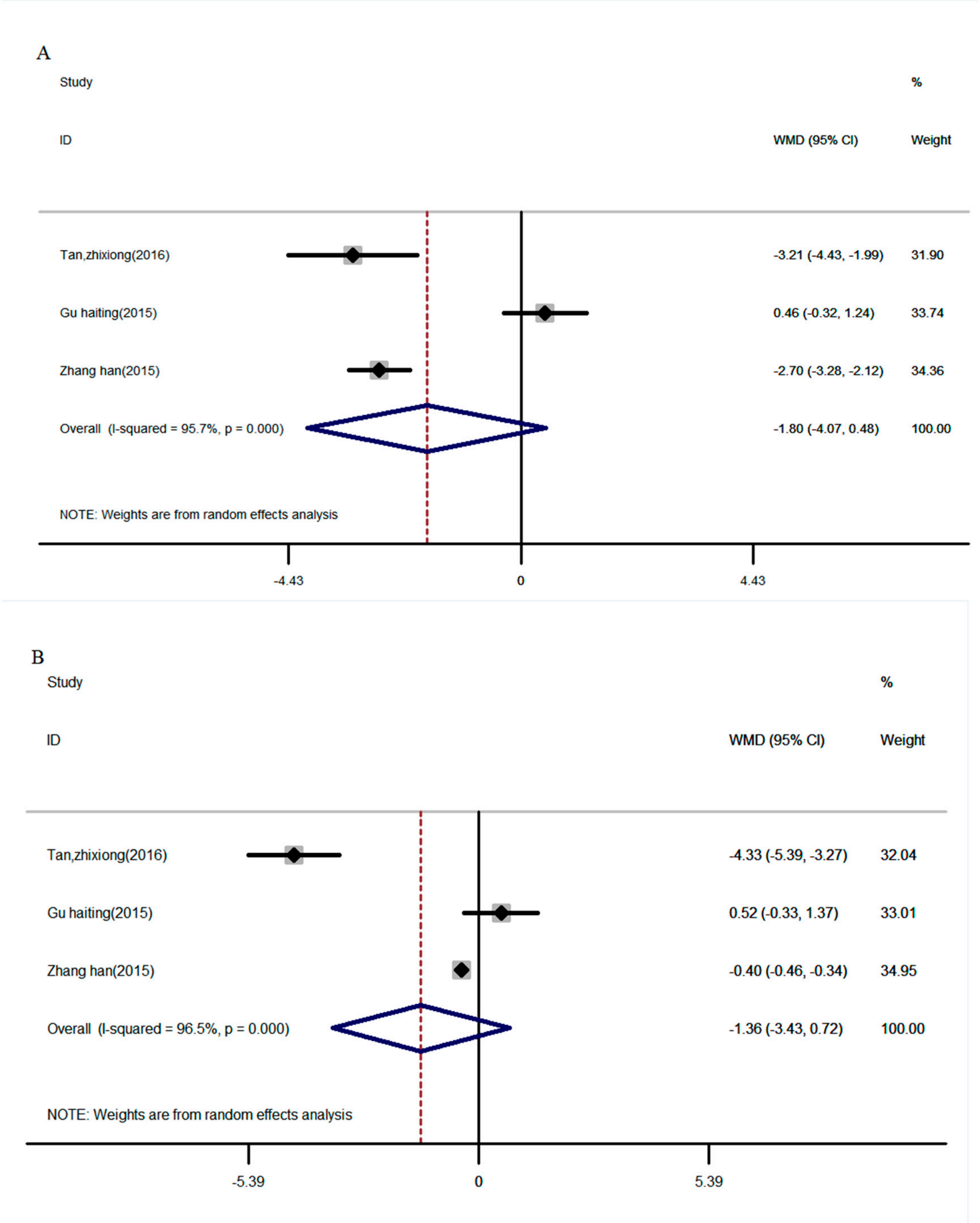

Supplementary Figure S4: Meta-analysis of vitamin D supplementation patients with COPD on $CD8^+$. C: differences of $CD8^+$ between baseline and post-intervention, D: $CD8^+$ of post-intervention, COPD, chronic obstructive pulmonary disease.

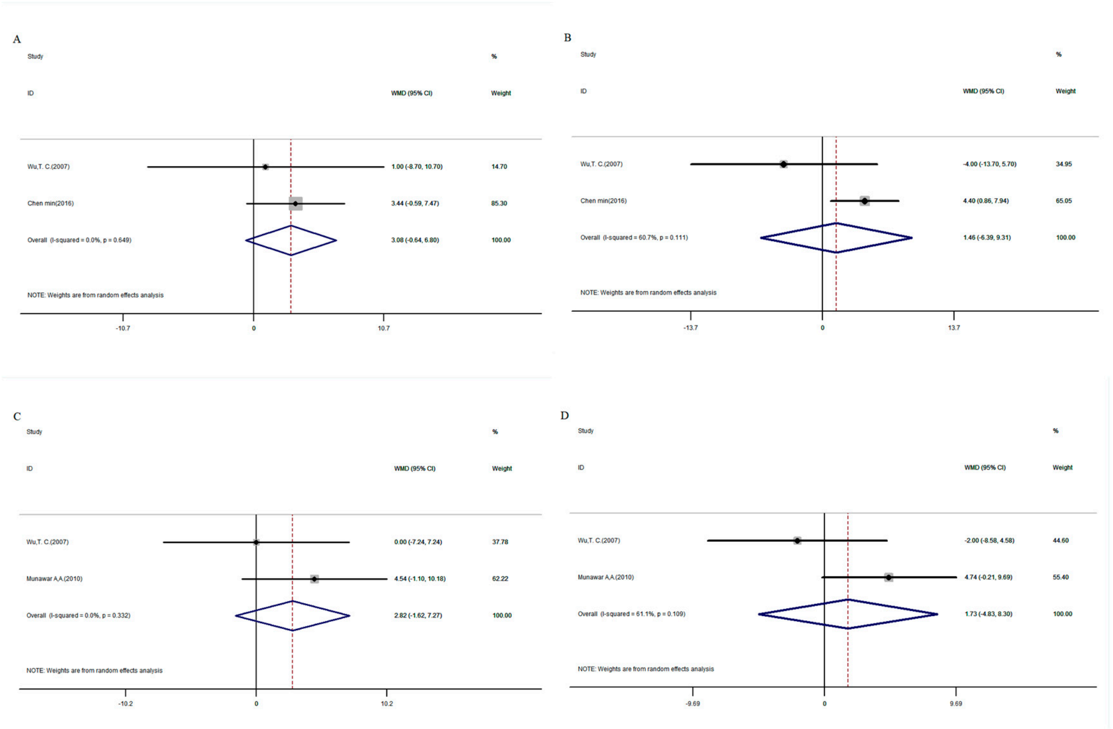

Supplementary Figure S5: Meta-analysis of vitamin C supplementation patients with COPD on FEV1 and FEV1/FVC. A: differences of FEV1 between baseline and post-intervention, B: FEV1 of post-intervention, C: differences of FEV1/FVC between baseline and post-intervention, D: FEV1/FVC of post-intervention. FEV1, forced expiratory volume in 1 second; FEV1/FVC, the ratio of forced expiratory volume in 1 second and forced vital capacity; COPD, chronic obstructive pulmonary disease.

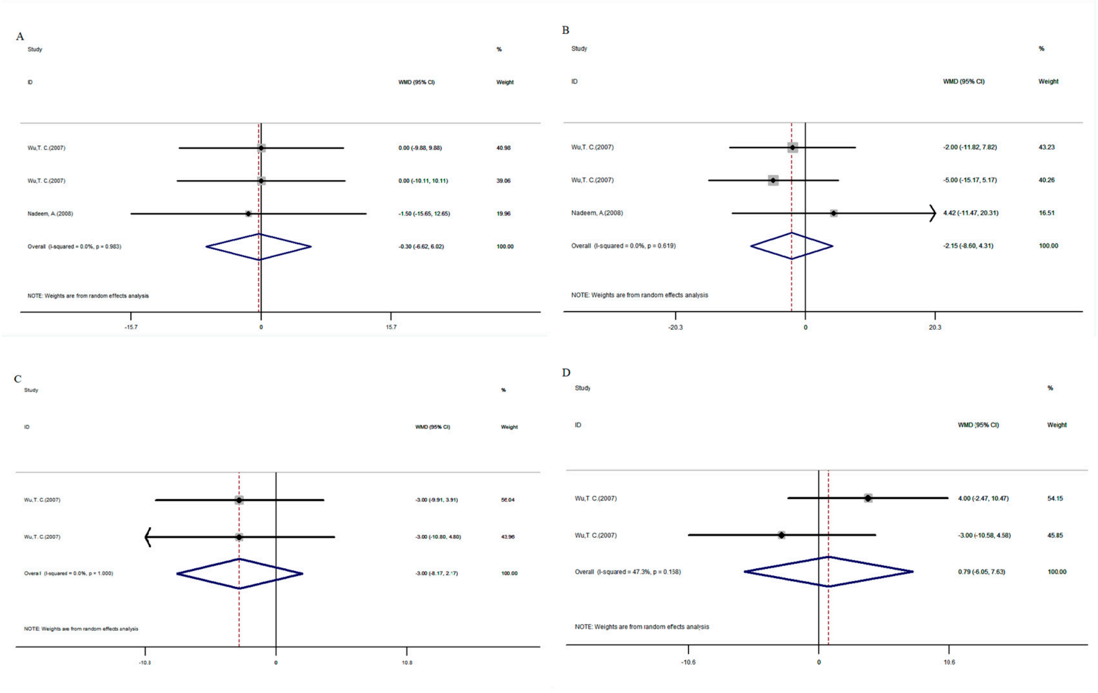

Supplementary Figure S6: Meta-analysis of vitamin E Supplementation patients with COPD on FEV1 and FEV1/FVC. A: differences of FEV1 between baseline and post-intervention, B: FEV1 of post-intervention, C: differences of FEV1/FVC between

baseline and post-intervention, D: FEV1/FVC of post-intervention. FEV1, forced expiratory volume in 1 second; FEV1/FVC, the ratio of forced expiratory volume in 1 second and forced vital capacity; COPD, chronic obstructive pulmonary disease.

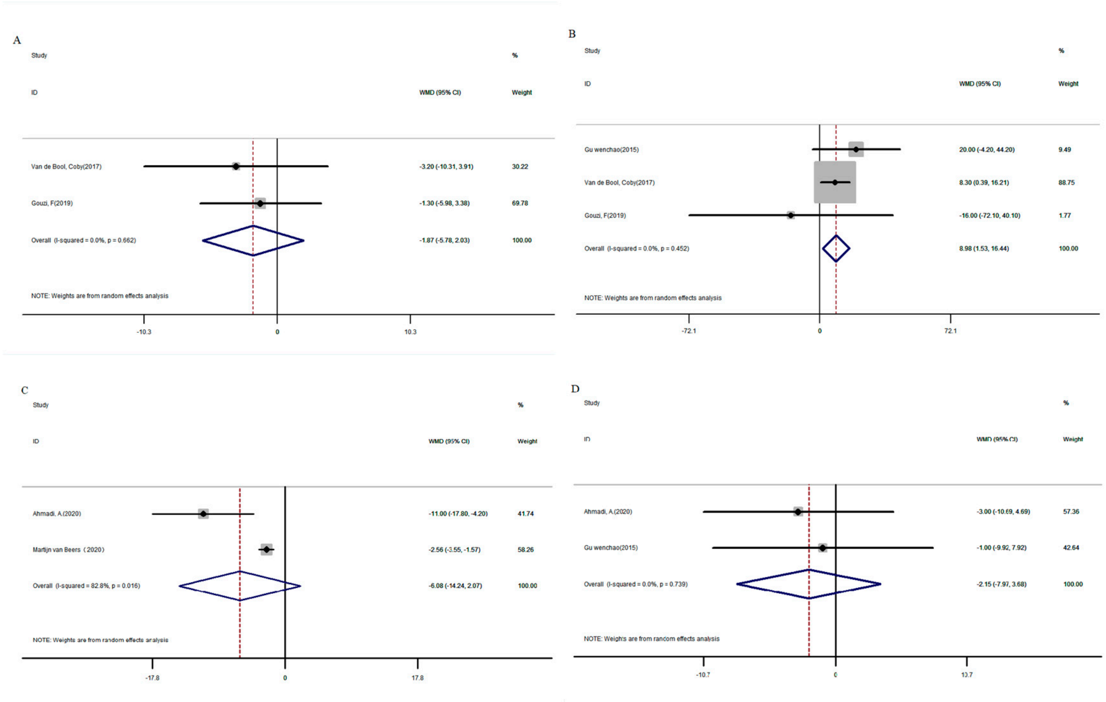

Supplementary Figure S7: Meta-analysis of compound nutrients supplementation patients with COPD on the score of 6WMD and SGRQ. A: differences of 6WMD between baseline and post-intervention, B: 6WMD of post-intervention, C: differences of SGRQ between baseline and post-intervention, D: SGRQ of post-intervention, 6MWD, 6-minute walk distance; SGRQ, St George's Respiratory Questionnaire, COPD, chronic obstructive pulmonary disease.
